# Prognostic Utility of Neck Lymph Node-to-Primary Tumor Standardized Uptake Value Ratio in Oral Cavity Cancer

**DOI:** 10.3390/biomedicines11071954

**Published:** 2023-07-11

**Authors:** Kuo-Wei Ho, Ku-Hao Fang, Chang-Hsien Lu, Cheng-Ming Hsu, Chia-Hsuan Lai, Chun-Ta Liao, Chung-Jan Kang, Yuan-Hsiung Tsai, Ming-Shao Tsai, Ethan I. Huang, Geng-He Chang, Chien-An Ko, Ming-Hsien Tsai, Yao-Te Tsai

**Affiliations:** 1 Department of Nuclear Medicine, Chang Gung Memorial Hospital, Chiayi 613016, Taiwan; kwhomd@gmail.com; 2 College of Medicine, Chang Gung University, Taoyuan 330036, Taiwan; kilikkilik121415@gmail.com (K.-H.F.); luchanghsien@gmail.com (C.-H.L.); scm00031@gmail.com (C.-M.H.); chiahsuan7092@gmail.com (C.-H.L.); liaoct@cgmh.org.tw (C.-T.L.); handneck@gmail.com (C.-J.K.); russell.tsai@gmail.com (Y.-H.T.); b87401061@gmail.com (M.-S.T.); ehuang.mdphd@gmail.com (E.I.H.); genghechang@gmail.com (G.-H.C.); wowkoann@gmail.com (C.-A.K.); b9302094@cgmh.org.tw (M.-H.T.); 3 Department of Otorhinolaryngology-Head and Neck Surgery, Chang Gung Memorial Hospital, Taoyuan 333423, Taiwan; 4 Department of Hematology and Oncology, Chang Gung Memorial Hospital, Chiayi 613016, Taiwan; 5 Department of Otorhinolaryngology-Head and Neck Surgery, Chang Gung Memorial Hospital, Chiayi 613016, Taiwan; 6 Department of Radiation Oncology, Chang Gung Memorial Hospital, Chiayi 613016, Taiwan; 7 Department of Diagnostic Radiology, Chang Gung Memorial Hospital, Chiayi 613016, Taiwan; 8 Department of Otorhinolaryngology-Head and Neck Surgery, Chang Gung Memorial Hospital, Kaohsiung 833253, Taiwan

**Keywords:** oral cavity cancer, biomarker, FDG PET, prognosis, nomogram

## Abstract

We investigated the prognostic utility of preoperative neck lymph node-to-primary tumor maximum standardized uptake value ratios (NTRs) in oral cavity squamous cell carcinoma (OSCC). We retrospectively reviewed the medical records of 141 consecutive patients who were diagnosed as having OSCC and had received fluorodeoxyglucose–positron emission tomography within 2 weeks prior to radical surgery between 2009 and 2018. To determine the optimal NTR cutoff, receiver operating characteristic analysis for overall survival (OS) was executed. The NTR’s prognostic value for disease-free survival (DFS) and OS were determined through Cox proportional hazards analysis and the Kaplan–Meier method. We determined the median (range) follow-up duration to be 35.2 (2.1–122.4) months. The optimal NTR cutoff was 0.273, and patients with a higher NTR (≥0.273) exhibited significantly worse DFS and OS (*p* = 0.010 and 0.003, respectively). A higher NTR (≥0.273) predicted poorer DFS (hazard ratio: 2.696, *p* = 0.008) and OS (hazard ratio: 4.865, *p* = 0.003) in multivariable analysis. We created a nomogram on the basis of the NTR, and it could accurately predict OS (concordance index: 0.774). Preoperative NTRs may be a useful prognostic biomarker for DFS and OS in patients with OSCC who have undergone surgery. NTR-based nomograms may also be helpful prognostic tools in clinical trials.

## 1. Introduction

Head and neck cancer (HNC) has been established as the sixth most common cancer globally, and oral cavity squamous cell carcinoma (OSCC) is the most common type of HNC [1]. Approximately 380,000 new cases of OSCC were documented in 2020, and its incidence is expected to increase by as much as 40% by 2040 [2]. Primary definitive chemoradiotherapy (CRT) or ablative surgery with or without adjuvant therapy has been widely recognized as the cornerstone of treatment for OSCC [3]. Cisplatin-based chemotherapy is a widely used regimen in both the definitive and adjuvant settings for patients with OSCC [4]. Cisplatin binds to reactive centers on purine residues, causing damage to the cancer cell’s deoxyribonucleic acid and inhibiting their division, ultimately leading to apoptosis of the OSCC cells [5]. Among extant prognostication tools for OSCC management, the tumor–node–metastasis (TNM) staging system is recognized to be the most extensively used. However, patients’ survival outcomes may differ, even if they share an identical TNM stage, with a possible explanation for this being the TNM system’s lack of key prognostic factors, such as perineural invasion (PNI) status [6], lymphovascular invasion (LVI) status [7], and poor differentiation (P-D) [8]. By incorporating prognostic stratifications that utilize clinical biomarkers in conjunction with the TNM staging system, treatment outcomes of OSCC can be enhanced through early diagnosis and the effective monitoring of treatment [9]. Nevertheless, the existing strategies primarily rely on the costly analysis of proteins or molecular factors derived from blood or saliva, and thus far, conclusive data regarding their effectiveness in OSCC management remains elusive [10,11]. Therefore, the identification of effective tumor-related prognostic biomarkers is of paramount importance to aid in patient stratification and to enable personalized treatment planning.

^18^F-fluorodeoxyglucose–positron emission tomography (^18^F-FDG PET) is extensively employed in pretreatment workup processes for OSCC [12], particularly in those with advanced-stage disease [13]. As described in the literature, in patients with HNC, the maximum standardized uptake value (SUVmax) derived for regional enlarged lymph nodes (SUV-N) and that derived for the primary tumor (SUV-T) exhibit prognostic utility [14,15]. Researchers have also demonstrated significant associations between the SUV-N-to-SUV-T ratio (NTR) and survival outcomes for assorted types of human cancer, such as lung cancer [16], cervical cancer [17], esophageal cancer [18], breast cancer [19], and nasopharyngeal cancer [20]. However, information regarding the prognostic utility of the NTR among patients with OSCC is lacking, which has prevented guidelines regarding the NTR’s clinical utility in the management of OSCC from being established. Given that the NTR reflects the metabolic activity of neck lymph nodes relative to primary OSCC, we hypothesized that the NTR would have prognostic value for survival outcomes in OSCC. This study aimed to determine whether preoperative NTR, measured through preoperative ^18^F-FDG PET, would have prognostic utility for patients with an OSCC diagnosis who had undergone curative surgery. To determine the NTR’s clinical applicability and forecast personalized survival outcomes, an NTR-based nomogram was constructed.

## 2. Materials and Methods

### 2.1. Patients and Data Extraction

Medical data on 156 consecutive patients with pathologically diagnosed primary OSCC who underwent preoperative ^18^F-FDG PET and radical surgery in our hospital between 2009 and 2018 were retrospectively reviewed. Patients who were determined to have a history of cancer or distant metastasis at presentation (*n* = 3), those who were subjected to neoadjuvant treatment before surgery (*n* = 2), those who had missing data pertaining to survival analysis (*n* = 9), and those who had acute infection within 1 month of surgery (*n* = 1) were excluded. Accordingly, data on 141 patients were subjected to formal analysis. This study was conducted in accordance with the Declaration of Helsinki. In addition, our applied study protocol was ratified by the hospital institutional review board, and owing to our study design’s retrospective nature, the necessity of obtaining informed consent was waived.

From our patients’ electronic clinical data on tumor location, we retrieved; age at OSCC diagnosis; sex; pathological stage based on the American Joint Committee on Cancer Staging Manual (8th edition, 2018); extranodal extension (ENE) status; tumor differentiation; PNI status; LVI status; closest resection margin status; and depth of invasion (DOI). All patients underwent preoperative workup processes, which comprised head and neck magnetic resonance imaging or computed tomography (CT), nuclear bone scanning, abdominal ultrasonography, and ^18^F-FDG PET 2 weeks or less before surgery. Furthermore, the Charlson comorbidity index (CCI) was applied to define and quantify underlying comorbidities [21]. Regarding personal habits, smoking and alcohol habits were defined as smoking of more than 10 cigarettes a day for 1 year or more [22] and consumption of at least 1 alcoholic drink a week for more than 6 months [23], respectively, and betel nut chewing habit was defined as consumption of one or more betel nuts a day for 1 year or more. We then categorized patients depending on whether they exhibited none, one, or at least two of these habits. All of the included patients were noted to undergo radical surgery that involved neck dissection. If indicated, adjuvant intensity-modulated radiotherapy (RT; dose: 2 Gy per fraction, 5 days a week; total dose: 60−66 Gy) or adjuvant CRT (delivered treatment: 40 mg/m^2^ of intravenous cisplatin once per week or 100 mg/m^2^ once every 3 weeks; total dose: 66 Gy) was rendered within 6 weeks of surgery in accordance with our institution’s guidelines [24].

### 2.2. SUV Measurement in ^18^F-FDG PET

Six-hour fasting blood sugar levels in all patients were measured before PET. If blood sugar was >200 mg/dL before the study, the PET was rescheduled. After 370 MBq of ^18^F-FDG was administered intravenously, we employed a lutetium-oxyorthosilicate-detector-equipped and 64-slice-CT-scanner equipped Biograph TruePoint 64 PET/CT scanner (Siemens, Knoxville, TN, USA) for image acquisition. To correct for PET attenuation, a low-dose unenhanced CT scan was executed, and, with regard to the parameters employed in this process, the tube voltage, current, rotation time, and pitch were set to 120 kV, 50 mAs, 0.5 s per rotation, and 0.8, respectively. True whole-body PET images were then taken from the vertex of the skull to the feet. The regions of interest (ROIs) on simultaneously displayed sagittal, coronal, and axial tomography images were determined to be those covering lesions exhibiting ^18^F-FDG uptake. An experienced nuclear medicine physician (Kuo-Wei Ho) delineated the ROIs that were used to calculate body-weight-normalized SUVs, and, in each ROI, SUVmax was assigned to the voxel with the greatest FDG uptake. We derived the NTR as the SUV-N-to-SUV-T ratio [25].

### 2.3. Follow-Up and Study Endpoints

Follow-up assessments were executed for all patients in the outpatient clinic at intervals of 1–2 months for the first year, at intervals of 3–4 months for the second and third years, and at intervals of 6 months for subsequent years. These assessments comprised flexible nasopharyngeal fiberscope and physical examinations executed during each visit, in addition to periodic imaging surveillance executed every 6–12 months. Our study endpoints comprised overall survival (OS)—described as the time between the radical surgery date and all-cause death, censor, or last follow-up dates—and disease-free survival (DFS), described as the time between the radical surgery and locoregional recurrence or metastasis, censor, or last follow-up dates. The final follow-up date was set to 31 December 2019.

### 2.4. Statistical Analysis

We executed the Kolmogorov–Smirnov test to assess the normality of our collected data. In addition, to derive the optimal NTR cutoff for predicting patient OS, receiver operating characteristic (ROC) curve analysis along with the Youden index was conducted [26]. Subsequently, we categorized patients into high- and low-NTR groups on the basis of the NTR cutoff value and compared their clinicopathological features through a chi-squared or Mann–Whitney *U* test. Using the Kaplan–Meier method, the study created plots of OS and DFS curves; moreover, between-group differences in terms of survival were determined through a log-rank test. Prognostic variables’ independent effects were analyzed using Cox proportional hazards analyses and are expressed herein using hazard ratios (HRs) with 95% confidence intervals (CIs). Prognostic factors exhibiting P values of <0.1 in the univariable analysis were then included in the multivariable analysis, and stepwise selection of the optimal subset of prognostic variables was performed in R 4.2.0. Two-sided *p* values of <0.05 indicated significant differences. Our study’s statistical analyses were all executed in SPSS (version 23; IBM, Armonk, NY, USA).

To evaluate the NTR’s clinical applicability, a nomogram incorporating the NTR and independent prognostic factors in the multivariable analysis was developed by employing the rms package (R version 5.1-0; Vanderbilt University, Nashville, TN, USA); the developed nomogram was applied to predict 3- and 5-year OS [27]. To determine the accuracy of the predictions, Harrell’s concordance values (C-index) were calculated for the TNM staging system (used for comparison) and for our nomogram. Finally, to ascertain whether predicted OS exhibited consistency with the actual survival outcomes, calibration plots for 3- and 5-year OS were also created.

## 3. Results

### 3.1. Baseline Characteristics

Table 1 presents the patients’ baseline characteristics. Men (121) were noted to constitute 85.8% of the 141 eligible patients. The study sample’s median (interquartile range (IQR)) age was determined to be 59 (51–67) years. In addition, more than 50% of the patients had stage IV OSCC (*n* = 92; 65.2%), and 48.2% had neck lymph node metastasis. Regarding the treatment modality, 48 (34.0%), 75 (53.2%), and 18 (12.8%) patients underwent surgery alone, surgery plus adjuvant CRT, and surgery plus adjuvant RT, respectively. The median (IQR) values SUV-N, SUV-T, and NTR values were 4.14 (3.02–6.91), 13.03 (8.02–18.31), and 0.388 (0.262–0.574), respectively.

### 3.2. Determination of Optimal NTR Cutoff Value

**Figure 1:**
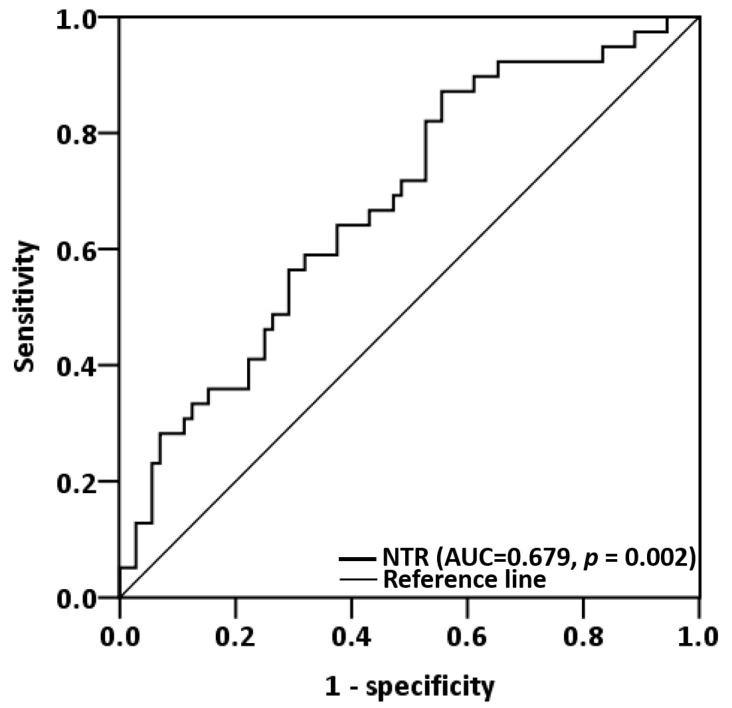
ROC analysis for OS prediction based on the NTR in 141 patients with OSCC. The AUC was 0.679 (*p* = 0.002; specificity = 44.4%; sensitivity = 87.2%), and 0.273 was determined to be the optimal NTR cutoff for OS prediction. Abbreviations: AUC, area under the curve; NTR, lymph node-to-primary tumor standardized uptake value ratio.

ROC analysis revealed the optimal NTR cutoff value for predicting OS to be 0.273 (area under the curve (AUC) = 0.679; 95% CI = 0.576−0.781; *p* = 0.002; Figure 1). The specificity and sensitivity at this value were 44.4% and 87.2%, respectively. Patients were categorized into high- (≥0.273; *n* = 103) and low-NTR (<0.273; *n* = 38) groups on the basis of the NTR cutoff value.

### 3.3. Associations between NTR and Clinicopathological Characteristics

Table 2 presents the clinicopathological characteristics of the high- (≥0.273) and low-NTR (<0.273) groups. The high-NTR group tended to be aged <65 years (*p* = 0.043), have neck lymph node metastasis (*p* = 0.016), display ENE (*p* = 0.002), and exhibit shorter median survival times (*p* = 0.004) than did the low-NTR group. Otherwise, no significant between-group differences in age, sex, cancer stage, T status, PNI, LVI, tumor differentiation, margin status, DOI, tumor location, treatment modality, CCI, or personal habits were observed.

### 3.4. Prognostic Factors for OS

**Figure 2:**
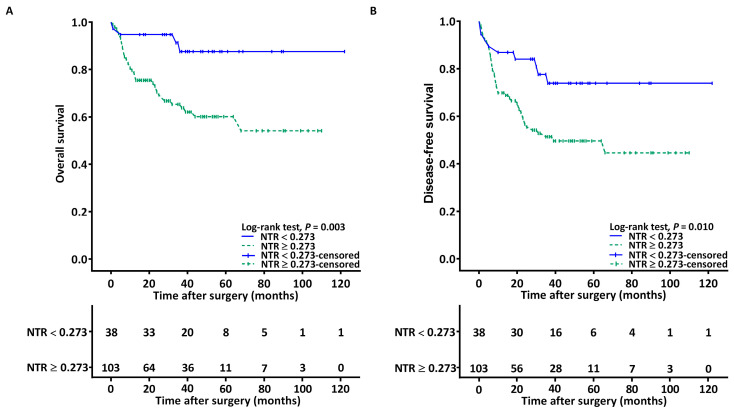
Kaplan–Meier curve of (**A**) OS and (**B**) DFS, stratified by the NTR. Worse prognosis was observed in patients with an NTR of ≥0.273. Abbreviation: NTR, lymph node-to-primary tumor standardized uptake value ratio.

We determined the median (IQR) follow-up duration for all patients to be 35.2 (14.2–51.1) months. The mean OS rates for the low- and high-NTR groups were estimated to be 110 (95% CI = 98–121) and 70 (95% CI = 60–81) months, respectively (log-rank *p* = 0.003; Figure 2A). Stage III–IV OSCC (*p* < 0.001), PNI (*p* = 0.001), LVI (*p* = 0.005), P-D (*p* = 0.005), the need for adjuvant therapy (*p* = 0.003), closest surgical margin < 5 mm (*p* = 0.024), and NTR ≥ 0.273 (*p* = 0.006; Table 3) exhibited significant associations with poor OS in the univariable analysis. The multivariable analysis revealed that stage III–IV disease (HR = 4.048; 95% CI = 1.785–20.878; *p* = 0.012), P-D (HR = 2.263; 95% CI = 1.129–4.946; *p* = 0.023), and NTR ≥ 0.273 (HR= 4.865; 95% CI = 1.704–13.887; *p* = 0.003) constituted risk factors for poor OS.

### 3.5. Prognostic Factors for DFS

The mean DFS rates for the low- and high-NTR groups were estimated to be 95 (95% CI = 79–110) and 59 (95% CI = 49–70) months, respectively (log-rank *p* = 0.010; Figure 2B). Table 4 presents the associations between DFS and the clinicopathological variables. The univariable analysis results revealed that stage III–IV disease (*p* < 0.001), the presence of PNI (*p* = 0.014), P-D (*p* = 0.028), the need for adjuvant therapy (*p* = 0.003), closest surgical margin < 5 mm (*p* = 0.031), and NTR ≥ 0.273 (*p* = 0.014) had substantial associations with poor DFS. The multivariable analysis revealed that NTR ≥ 0.273 (HR = 2.696; 95% CI = 1.301–5.586; *p* = 0.008) and stage III–IV disease (HR = 2.725; 95% CI = 1.269–5.896; *p* = 0.021) constituted risk factors for poor DFS.

### 3.6. Construction of Predictive Nomogram

**Figure 3:**
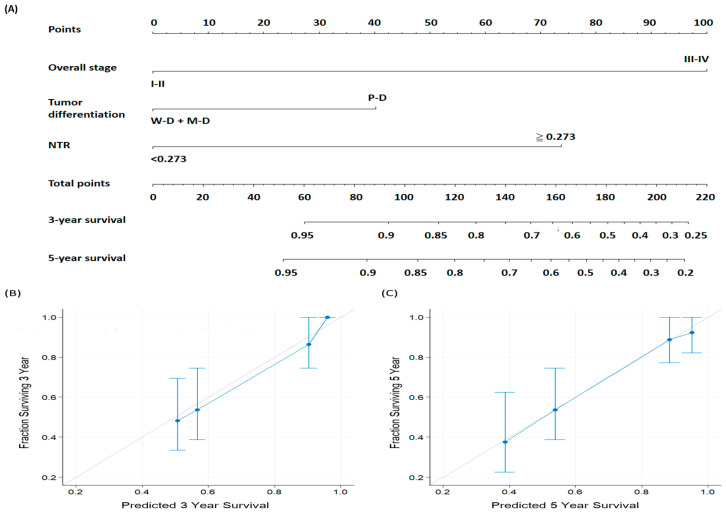
(**A**) Nomogram for OS prediction incorporating independent prognostic factors in the multivariable analysis. Each prognostic factor’s contribution to risk is represented by the line segments and uppermost points. Total score is the sum of each factor’s points. Drawing a vertical line downward from the total score point yields the likelihood of 3- and 5-year OS. Calibration plots for (**B**) 3-year OS and (**C**) 5-year OS. The gray line at 45° represents perfect OS prediction; the predicted outcomes of the nomogram are represented by the blue line. The blue dots and bars reflect the performance of the nomogram and the 95% CIs for the OS predictions, respectively. Abbreviations: MD, moderately differentiated; NTR, lymph node-to-primary tumor standardized uptake value ratio; PD, poorly differentiated; WD, well differentiated.

This study created a predictive nomogram that comprised independent prognosticators in the multivariable analysis, namely, cancer stage, tumor differentiation, and NTR (Figure 3). The nomogram’s AUC was 0.78 (cutoff: 157; sensitivity: 82.9%; specificity: 67.5%). The C-index value derived for the nomogram based on the AJCC system was 0.68 (95% CI = 0.64–0.72) and that derived for the nomogram based on the NTR was 0.77 (95% CI = 0.73–0.82). Furthermore, the calibration plots demonstrated that the nomogram-predicted rates of 3- and 5-year OS exhibited consistency with the observed rates (Figure 3B and 3C, respectively). According to these results, our nomogram demonstrates the NTR’s clinical applicability and is feasible for use in predicting personalized OS in OSCC management.

## 4. Discussion

We determined, on the basis of our literature review, that our current study is the first to research the prognostic utility of ^18^F-FDG PET metabolic activity in neck lymph nodes relative to that in primary tumors in patients with OSCC. By assessing clinical data collected from OSCC cohorts that underwent similar treatment protocols, we yielded several key results. An NTR of ≥0.273 derived preoperatively exhibited an association with adverse pathological features of OSCC, namely neck nodal metastasis and ENE. DFS and OS significantly differed after patient stratification by NTR (*p* = 0.010 and 0.003, respectively). As demonstrated by our executed multivariable analysis, NTRs of ≥0.273 were independent factors predicting poor DFS and OS. Several pathological features of OSCC, such as PNI and LVI, did not exhibit independent prognostic significance in our study, suggesting that the ability to predict a prognosis solely on the basis of these factors is limited. C-index values and the calibration plots reveal that the nomogram based on the NTR accurately predicted OS, suggesting that the NTR has an informative role in OSCC prognostication. The findings of this study may serve as reference for clinicians, helping them to preemptively identify patients with OSCC with unfavorable treatment outcomes and devise intensive treatment plans and surveillance strategies.

Although we demonstrated that a high NTR value represented an independent prognostic factor for adverse OS and DFS in a population comprising patients with OSCC who had undergone surgery, the mechanism underlying these correlations remains uncertain. A high NTR would indicate an increase in SUV-N and/or a decrease in SUV-T; therefore, the prognostic effects of the components of the NTR—SUV-T and SUV-N—may provide some clues. SUV-N is useful in the detection of neck metastatic lymphadenopathy in patients with OSCC who do not exhibit palpable neck masses [28]. Hung et al. have revealed that the SUVs of both lymph nodes and tumors were independent prognostic factors for patients who had nasopharyngeal carcinoma [15]. A retrospective study reviewing 257 patients with OSCC identified that SUV-N ≥ 15.9 and levels IV and V metastasis in the neck were independent risk factors for adverse OS, disease-specific survival, distant-metastasis-free survival (DMFS), and DFS [29]. High values for SUV-T, the denominator of the NTR, were correlated with adverse pathological features of OSCC, such as greater DOI, PNI, tumor grade, and bone infiltration [12,30], and higher SUV-T values predicted occult neck lymph node metastasis in patients diagnosed as having OSCC [31]. However, the prognostic value of SUV-T is inconsistent within the literature. For example, Suzuki et al. reported that SUV-T ≥ 12 exhibited a significant correlation with poorer 3-year OS and DMFS in OSCC [32]. Conversely, researchers have reported that pretreatment total lesion glycolysis and metabolically active tumor volume are independent predictors of OS and metastasis in HNC, whereas SUV-T is not [33,34,35]. In addition, researchers elucidated the associations between the NTR and clinicopathological features in different types of cancer, providing clues regarding the mechanism through which the NTR can predict cancer prognosis. Chung et al. retrospectively studied 107 patients diagnosed as having endometrial cancer and reported significant associations between a high preoperative NTR and tumor recurrence, advanced cancer stage, LVI, lymph node metastasis, deep myometrial infiltration, and tumor grade [36]. In esophageal cancer, a high NTR of >0.46 has been associated with advanced nodal status rather than tumor differentiation, size, or location [18]. Our results indicate that a high NTR of ≥0.273 tends to be associated with cervical metastatic lymphadenopathy and ENE, suggesting that preoperative relative metabolic activity on PET scans can be a surrogate indicator of OSCC aggressiveness, particularly neck nodal metastasis. The results of the abovementioned studies suggest a potential mechanism through which elevated NTRs may be associated with poor OSCC prognosis. However, the potential prognostic benefits of the NTR rather than SUV-N or SUV-T alone remain to be identified. Various factors can interfere with the interpretation of ^18^F-FDG PET results in head and neck imaging [37], and we suggest that the NTR may prevent the interference of these factors, such as uncertainties in tracer dose, patients’ body weight, acquisition time, and machine calibration [18], in the quantitation of SUV and that it provides internal control, as do other ratio methods [38]. Additionally, we suggest that the NTR may reflect the metastatic potential of OSCC. A high NTR may indicate higher metabolic activity in metastatic lymph nodes than in primary tumors, suggesting that the risk of locoregional and distant metastasis is high, which may result in worse survival outcomes. Further study is needed to explore the mechanism through which the NTR is associated with survival outcomes in OSCC.

Studies have explored the NTR’s prognostic value in several cancer types. After reviewing 437 patients with nasopharyngeal carcinoma, Hung et al. discovered that an NTR of >0.9181 had a significant correlation with lower DMFS in patients with N2–3 status and stage IV disease and that it represented an independent DMFS prognostic factor (HR: 2.20, *p* = 0.011) [25]. In addition, Chung et al. explored the NTR’s prognostic utility in uterine cervical cancer, reporting that an NTR of >0.1747 independently predicted decreased progression-free survival (HR = 11.358, *p* = 0.048) [17]. By retrospectively evaluating clinical data on 119 patients with invasive ductal breast cancer, Kim et al. discovered that the NTR, rather than SUV-T or SUV-N, is a significant independent factor predicting relapse in breast cancer [19]. Chen et al. conducted a retrospective study on 96 patients diagnosed as having unresectable esophageal squamous cell carcinoma and revealed that an NTR of >0.46 represented an independent risk factor for worse DMFS (HR: 1.81, *p* = 0.023) and OS (HR: 1.77, *p* = 0.014) [18]. Although the results of these studies are consistent with ours and support the prognostic role of the NTR in survival outcomes in various types of cancer, these studies were retrospective, and the majority of these studies enrolled relatively small study populations. Accordingly, additional large-scale prospective studies are warranted to confirm the NTR’s prognostic role, particularly in early-stage cancer.

We determined, on the basis of our literature review, that our executed research is the first to create a nomogram based on PET parameters for patients with OSCC. The nomogram demonstrates the NTR’s clinical applicability and can accurately predict 3- and 5-year OS. Several studies have investigated the predictive abilities of a PET-parameter-based nomogram for other types of cancer and reported similar findings. In a study on 76 patients diagnosed as having locally advanced oropharyngeal cancer, Castelli et al. developed a nomogram based on the metabolic tumor volume of the lymph nodes and primary tumors; their nomogram was noted to accurately predict locoregional control and OS [39]. Xu et al. constructed a nomogram incorporating SUV-N and pathological T status and reported that it performed well in predicting nodal metastasis in patients with cN0 OSCC [40]. In a study involving 234 patients with cervical cancer, Kidd et al. also created a nomogram based on lymph node status in FDG PET scans, SUV-T, and tumor volume and revealed that the nomogram performed satisfactorily in predicting OS, recurrence-free survival, and disease-specific survival [41]. These study results support the use of NTR-based nomograms in OSCC management, which can then be considered in future cancer research and clinical practice.

The NTR’s clinical implications include the potential prognostic stratification of OSCC before surgery. This ratio has potential to facilitate the identification of patients with a high risk of treatment failure, as these patients may benefit from more personalized treatment and intensive surveillance. Furthermore, the interpretation of ^18^F-FDG PET results can be influenced by various factors, which inherently imposes limitations on the use of SUV as a standalone parameter. By integrating both SUV-N and SUV-T, the NTR may provide a solution to address the limitations of relying solely on SUV as a prognostic parameter for OSCC. Finally, the constructed nomogram demonstrates the clinical feasibility of the NTR and effectively provides accurate predictions of OS. Our study involves several limitations that must be acknowledged. First, its single-institute, retrospective design constitutes a major limitation that may have resulted in inherent selection bias. Second, the results were not validated using independent cohorts from different centers, which limits their generalizability. In addition, no consensus was reached in the literature with regard to the optimal NTR cutoff for OSCC prognostication. Although the derived cutoff of 0.273 from the ROC analysis demonstrated statistical significance in survival analysis, the corresponding AUC value of 0.679 is indicative of less favorable performance. Consequently, it is crucial to approach the interpretation and application of our findings with caution. Finally, a few patients with stage I and II disease were enrolled because ^18^F-FDG PET scans are infrequently conducted for patients with early-stage OSCC; this may have resulted in an underestimation of the NTR’s prognostic value in patients with early-stage disease. Hence, before the NTR can be clinically applied, large-scale prospective studies should be executed to support our results. The NTR’s prognostic value in patients with early-stage OSCC and those undergoing nonsurgical treatment for OSCC also warrants further investigation.

## 5. Conclusions

The results indicate that NTR values measured using preoperative ^18^F-FDG PET scans are a convenient and valuable prognosticator for DFS and OS among patients with surgically treated OSCC. NTR-based nomograms can accurately predict survival and should thus be considered in oncological practice and research.

## Figures and Tables

**Table 1:** Baseline characteristics of 141 patients.

Characteristic	Numbers (%)
Age (years), median (IQR)	59 (51−67)
Sex	
Men	121 (85.8%)
Women	20 (14.2%)
Tumor location	
Buccal mucosa	51 (36.2%)
Tongue	45 (31.9%)
Gingiva	28 (19.9%)
Retromolar trigone	9 (6.4%)
Mouth floor	4 (2.8%)
Lip	2 (1.4%)
Hard palate	2 (1.4%)
Personal Habits	
Cigarette Smoking	118 (83.7%)
Betel nut chewing	116 (82.3%)
Alcohol consumption	100 (70.9%)
AJCC stage	
I	13 (9.2%)
II	17 (12.1%)
III	19 (13.5%)
IV	92 (65.2%)
T status	
T1	19 (13.5%)
T2	25 (17.7%)
T3	22 (15.6%)
T4	75 (53.2%)
N status	
N0	73 (51.8%)
N1	12 (8.5%)
N2	45 (31.9%)
N3	11 (7.8%)
Presence of PNI	48 (34.0%)
Presence of ENE	43 (30.5%)
Presence of LVI	12 (8.5%)
Tumor differentiation	
WD/MD	121 (85.8%)
PD	20 (14.2%)
Closest surgical margin	
≥5 mm	110 (78.0%)
<5 mm	31 (22.0%)
DOI ≥ 10 mm	87 (61.7%)
Treatment modality	
Surgery alone	48 (34.0%)
Surgery + RT	18 (12.8%)
Surgery + CRT	75 (53.2%)
Charlson comorbidity index	
0	82 (58.2%)
≥1	59 (41.8%)
SUV-T, median (IQR)	13.03 (8.02−18.31)
SUV-N, median (IQR)	4.14 (3.02−6.91)
NTR, median (IQR)	0.388 (0.262−0.574)

Abbreviations: AJCC, American Joint Committee on Cancer; CRT, chemoradiotherapy; DOI, depth of invasion; ENE, extranodal extension; IQR, interquartile range; LVI, lymphovascular invasion; MD, moderately differentiated; NTR, lymph node-to-primary tumor standardized uptake value ratio; PD, poorly differentiated; PNI, perineural invasion; RT, radiotherapy; SUV-N, the highest standardized uptake value of neck lymph nodes; SUV-T, the highest standardized uptake value of primary tumor; WD, well differentiated.

**Table 2:** Clinicopathological characteristics according to the cutoff of NTR.

	Number of Patients (%)		
Characteristic	NTR < 0.273, *n* = 38	NTR ≥ 0.273, *n* = 103	*p*-Value
Sex			0.194 ^a^
Men	35 (92.1%)	86 (83.5%)	
Women	3 (7.9%)	17 (16.5%)	
Age			0.043 ^a^
<65	20 (52.6%)	73 (70.9%)	
≥65	18 (47.4%)	30 (29.1%)	
AJCC Stage			0.935 ^a^
I–II	13 (34.2%)	36 (35.0%)	
III–IV	25 (65.8%)	67 (65.0%)	
T status			0.114 ^a^
T1–T2	8 (21.1%)	36 (35.0%)	
T3–T4	30 (78.9%)	67 (65.0%)	
N status			
N0	26 (68.4%)	47 (45.6%)	0.016 ^a^
N1–N3	12 (31.6%)	56 (54.4%)	
PNI			
Absent	26 (68.4%)	67 (65.0%)	0.708 ^a^
Present	12 (31.6%)	36 (35.0%)	
LVI			
Absent	37 (97.4%)	92 (89.3%)	0.181 ^a^
Present	1 (2.6%)	11 (10.7%)	
ENE			0.002 ^a^
Absent	34 (89.5%)	64 (62.1%)	
Present	4 (10.5%)	39 (37.9%)	
Tumor differentiation			0.832 ^a^
WD/MD	33 (86.8%)	88 (85.4%)	
PD	5 (13.2%)	15 (14.6%)	
Closest margin			0.871 ^a^
≥5 mm	30 (78.9%)	80 (77.7%)	
<5 mm	8 (21.1%)	23 (22.3%)	
DOI ≥ 10 mm			0.165 ^a^
No	11 (28.9%)	43 (41.7%)	
Yes	27 (71.1%)	60 (58.3%)	
Tumor location			0.704 ^a^
Tongue	12 (31.6%)	33 (32.0%)	
Buccal mucosa	12 (31.6%)	39 (37.9%)	
Other	14 (36.8%)	31 (30.1%)	
Personal habits			0.791 ^a^
No exposure	3 (7.9%)	12 (11.7%)	
One exposure	2 (5.3%)	6 (5.8%)	
Two or all exposure	33 (86.8%)	85 (82.5%)	
Adjuvant therapy			0.228 ^a^
Absent	15 (39.5%)	33 (32.0%)	
RT	7 (18.4%)	11 (10.7%)	
CRT	16 (42.1%)	59 (57.3%)	
CCI			0.239 ^a^
0	22 (57.9%)	60 (58.3%)	
≥1	16 (42.1%)	43 (41.7%)	
Survival in months, median (IQR)	40.5 (30.5–58.0)	29.0 (12.0–48.3)	0.004 ^b^

Abbreviations: CCI, Charlson comorbidity index; CRT, concurrent chemoradiotherapy; DOI, depth of invasion; ENE, extranodal extension; IQR, interquartile range; MD, moderately differentiated; NTR, lymph node-to-primary tumor standardized uptake value ratio; PNI, perineural invasion; PD, poorly differentiated; RT, radiotherapy; W−D, well differentiated. ^a^ Chi-square test; ^b^ Mann–Whitney U test.

**Table 3:** Univariable and multivariable analyses of clinical variables for overall survival.

Characteristic		Univariable Analysis		Multivariable Analysis	
		HR (95% CI)	*p*-Value	HR (95% CI)	*p*-Value
Sex	Men vs. Women	1.026 (0.401–2.622)	0.958		
Age (years)	≥65 vs. <65	0.745 (0.380–1.460)	0.391		
AJCC stage	III–IV vs. I–II	8.431 (2.600–27.338)	<0.001	4.048 (1.785–20.878)	0.012
Presence of PNI	Yes vs. no	2.863 (1.541–5.321)	0.001	1.609 (0.821–3.156)	0.166
Presence of LVI	Yes vs. no	3.273 (1.444–7.418)	0.005	1.570 (0.658–3.742)	0.309
Cancer histologic grading	PD vs. WD/MD	2.701 (1.347–5.415)	0.005	2.263 (1.129–4.946)	0.023
Adjuvant therapy	Yes vs. no	3.685 (1.547–8.781)	0.003	1.462 (0.542–3.944)	0.453
Closest margin (mm)	<5 vs. ≥5	2.103 (1.102–4.014)	0.024	1.508 (0.761–2.990)	0.239
CCI	≥1 vs. 0	1.513 (0.644–3.554)	0.342		
NTR	≥0.273 vs. <0.273	4.219 (1.501–11.854)	0.006	4.865 (1.704–13.887)	0.003

Abbreviations: AJCC, American Joint Committee on Cancer; CCI, Charlson comorbidity index; CI, confidence interval; HR, hazard ratio; LVI, lymphovascular invasion; MD, moderately differentiated; NTR, lymph node-to-primary tumor standardized uptake value ratio; PD, poorly differentiated; PNI, perineural invasion; WD, well differentiated.

**Table 4:** Univariable and multivariable analyses of clinical variables for disease-free survival.

Characteristic		Univariable Analysis		Multivariable Analysis	
		HR (95% CI)	*p*-Value	HR (95% CI)	*p*-Value
Sex	Men vs. Women	1.199 (0.513–2.800)	0.675		
Age (years)	≥65 vs. <65	0.534 (0.292–0.977)	0.042		
AJCC stage	III–IV vs. I–II	3.717 (1.822–7.586)	<0.001	2.725 (1.269–5.896)	0.021
Presence of PNI	Yes vs. no	1.923 (1.139–3.247)	0.014	1.216 (0.681–2.173)	0.509
Presence of LVI	Yes vs. no	2.054 (0.930–4.540)	0.075	1.237 (0.534–2.866)	0.620
Cancer histologic grading	PD vs. WD/MD	2.002 (1.076–3.724)	0.028	1.819 (0.944–3.505)	0.074
Adjuvant therapy	Yes vs. no	2.693 (1.392–3.687)	0.003	1.575 (0.692–3.584)	0.279
Closest margin (mm)	<5 vs. ≥5	1.850 (1.058–3.236)	0.031	1.453 (0.810–2.606)	0.210
CCI	≥1 vs. 0	1.146 (0.529–2.481)	0.730		
NTR	≥0.273 vs. <0.273	2.459 (1.204–5.023)	0.014	2.696 (1.301–5.586)	0.008

Abbreviations: AJCC, American Joint Committee on Cancer; CCI, Charlson comorbidity index; CI, confidence interval; HR, Hazard ratio; LVI, lymphovascular invasion; MD, moderately differentiated; NTR, lymph node-to-primary tumor standardized uptake value ratio; PD, poorly differentiated; PNI, perineural invasion; WD, well differentiated.

## Data Availability

The data sets generated for this study are available on request from the corresponding author.
